# Does swimming at the bottom serve as a hydraulic advantage for benthic fish *Neogobius melanostomus* Pallas (1814) in flowing water?

**DOI:** 10.1242/bio.060533

**Published:** 2024-10-30

**Authors:** Nandhakumar Govindasamy, Georg Rauter, Frank Seidel, Patricia Burkhardt-Holm, Philipp E. Hirsch, Joschka Wiegleb

**Affiliations:** ^1^ Program Man-Society-Environment, Department of Environmental Sciences, University of Basel, Vesalgasse 1, 4051 Basel, Switzerland; ^2^ BIROMED-Lab, Department of Biomedical Engineering, University of Basel, Gewerbestrasse 14, 4123 Allschwil, Switzerland; ^3^ Institute for Water and River Basin Management, Karlsruhe Institute of Technology, Kaiserstrasse 12, 76131 Karlsruhe, Germany; ^4^ Swiss University of Applied Sciences (PH FHNW), Bahnhofstrasse 6, 5210 Windisch, Switzerland

**Keywords:** Round goby, Fish, Hydraulic force, Artificial neural networks

## Abstract

Benthic fish, such as the round goby (*Neogobius melanostomus* Pallas, 1814) tend to swim near the bottom, especially at increased water velocities. To test whether these fish have a hydraulic advantage from swimming near the bottom and how the substrate affects the forces experienced, we measured the hydraulic forces experienced by preserved fish in a flow channel. The fish were tested 5.0 mm above the bottom at smooth and rough surface, and in the water column (10.0 cm elevation) above smooth and rough surface at 0.95 m/s water velocity. No significant effect among the mean hydraulic forces was observed between both fish positions, whereas the mean hydraulic forces in the water column were significantly higher (*P*<0.05) above the rough surface (mean 0.077 N±0.025 s.d.) than above the smooth surface (mean 0.068 N±0.021 s.d.). A convolutional neural network (CNN) predicted the column smooth treatment was the most characteristic force data time series (mean F1=0.88±0.03 s.d.). We conclude that the body posture and body movements of the fish are more relevant for the hydraulic forces experienced by the fish than the vertical position in the water column. Further factors explaining the affinity to swimming near the bottom are discussed.

## Teaser

**Summary:** The vertical position seems to be less relevant for the hydraulic forces that benthic fish experience in flowing water than body posture and fish movement.

## INTRODUCTION

Flowing water affects aquatic organisms in riverine habitats in multiple ways (Trinci et al., 2017). Turbulence can have destabilizing effects on swimming fish (Lupandin, 2005; Tritico and Cotel, 2010), but can also have supportive effects as observed by passive propulsion at specific flow conditions (Beal et al., 2006; Liao et al., 2003). When swimming in flowing water, fish exploit energetically supportive flow conditions to save energy (Facey and Grossman, 1992) and can make use of microhabitats (Facey and Grossman, 1992). Some benthic fish implemented this interaction with bottom hydraulics to save energy in their swimming styles and can hold positions across a variety of flow conditions without a change in oxygen consumption (Facey and Grossman, 1992).

Benthic stream fishes often use areas near the substrate that exhibit reduced or turbulent flow (Meyers and Belk, 2014), and swimming speed studies reported benthic fish as ‘weak’ swimmers compared to long-distance migrators, such as salmoniformes (Tudorache et al., 2008). The boundary layers used by benthic fish allow for escaping the flow (Carlson and Lauder, 2011). Some benthic fish implemented the usage of these boundary layers in their swimming style and developed a burst-and-hold swimming style (Egger et al., 2021; Gilbert et al., 2016). When staying at the bottom, some benthic fish use their pectoral fins to create forces directed to the bottom, which supports station holding with minimal effort while staying within the flow (Carlson and Lauder, 2011; Wiegleb et al., 2020). Nevertheless, to pass fields of increased flow, it may transpire that benthic fish must leave the sheltering layer near the bottom substrate and swim upstream through areas with increased flow. Because the fish rest at the bottom with minimum energetic effort, they can rest and wait for supportive flow conditions.

The energetic costs of swimming are related to fish morphology (Ohlberger et al., 2006). For instance, phenotypic adaptation between low- and high-velocity habitats was observed in the blacktail shiner *Cyprinella venusta*, a small cyprinid fish (Franssen et al., 2013). North American darters profit from small body size, which allows for escaping the flow and supports benthic station holding (Carlson and Lauder, 2011). A functional link between the shape of fish bodies and the velocity used by fish while swimming was observed by Sagnes and Statzner (2009).

Some benthic fish have a fusiform shape with high drag and low lift force (Webb, 1989). Whether these fish have a hydraulic advantage if swimming near the bottom in flowing water is presently unknown. Such insight would improve the understanding of benthic fish swimming behaviour in riverine habitats and allow for species-oriented restoration measures of rivers. The need to recover and restore degraded ecosystems, as well as the promotion of endangered species, is requested by Article 8 (f) of the Convention on Biological Diversity (UN, I, 1992).

The round goby is a prominent invasive benthic fish species native to the Ponto-Caspis (Kornis et al., 2012). Since the 1990s, the round goby has expanded its range in the Great Lakes watershed and European water bodies through translocation by commercial ships’ ballast water (Kornis et al., 2012; Wonham et al., 2000; Kotta et al., 2016; Johansson et al., 2018), recreational boats (Minchin et al., 2006; Rothlisberger et al., 2010; Murray et al., 2011; Bussmann and Burkhardt-Holm, 2020; Bussmann et al., 2022) and moving upstream in streams and tributary rivers via active swimming (Pennuto and Rupprecht, 2016). The round goby was reported to swim predominantly near the bottom, as observed during U_crit_ swimming performing tests (Egger et al., 2021). Following the fish body shape categorization from Webb (1989), the round goby is a fusiform body-shaped benthic fish. Several studies support that the round goby likely profits from the reduced flow velocity between rocks, substrates, and boundary layers in habitats such as tributaries, where swimming becomes even more challenging (Hoover et al., 2003; Pennuto and Rupprecht, 2016; Gilbert et al., 2016). Gravel or cobble structures are preferred habitats for invasive round gobies (Reid, 2019), potentially because they offer refugia that promote rest-and-coast or rest-and-burst behaviours, as described by Gilbert et al. (2016). Round gobies may alter their swimming mode to local flow conditions, and it is suggested that this behaviour could enable them to overcome barriers and support their dispersal into upstream tributaries (Wiegleb et al., 2020). There have been studies to prevent round goby migration using the selective barrier or fish passage technologies that facilitate the passage of native species without aiding the dispersal of non-natives, and these studies were conducted above smooth surfaces (Webb, 1989; Hoover et al., 2003; Haro et al., 2004; Tierney et al., 2011; Kerr et al., 2021; Wiegleb, 2022; Wiegleb et al., 2022). Generally, these studies concluded that the combination of increased velocity and smooth surface were found to act as a barrier and prevent round goby migration by impeding station holding and resting at the bottom. Thus, the round goby's recovery from the exertion of swimming is inhibited and, in consequence, the fish's swimming capacity is exceeded.

However, these studies looked into the live fish swimming behaviour, such as round goby using pelvic fins to hold the substrate and perform burst-and-hold swimming strategies, i.e. smooth substrates or surfaces can minimize opportunities for round goby to rest and recover when attempting to use burst-and-hold to navigate upstream in the riverine (Hoover et al., 2003; Haro et al., 2004; Tierney et al., 2011; Kerr et al., 2021; Wiegleb et al., 2022). As a fusiform fish species, the round goby is able to remarkably modulate the hydraulic forces experienced by its pectoral fins (Wiegleb et al., 2020). The pectoral fins can be adjusted in a way that the fish body experiences a negative lift force, which allows the round goby to rest, even at higher water-flow velocities (Carlson and Lauder, 2011; Wiegleb et al., 2023a).

In the present study, we explored whether an invasive benthic fish (round goby, *Neogobius melanostomus* Pallas, 1814) has a hydraulic advantage in an artificial flow channel from being near the bottom compared to 10 cm above. To account for the effect of bottom roughness on the hydraulics experienced by the round goby in flowing water, we performed the experiments for two bottom treatments: one treatment was represented by a smooth plexiglass plate, the other by a rougher bottom surface with gravel particles attached to the bottom. The mean hydraulic forces experienced by the fish were then compared between treatments statistically. In addition, we compared the temporal proceedings of the force measurement time series between treatments using artificial neural networks to account for temporal variation in the hydraulics affecting the preserved fish. Because of the affinity of benthic fish to swim near the bottom, their ability to profit energetically from interactions with the bottom substrate, and the reduction of the velocity near the bottom substrate (Bonakdari et al., 2008), we assumed that benthic fish have a general hydraulic advantage and experience smaller hydraulic forces when swimming near the bottom in flowing water. The convolutional neural network (CNN) was expected to achieve good prediction performance if characteristic patterns within the time series of the hydraulic forces were present among treatments, when trained to predict the treatments on the force data.

## RESULTS

### Fish shape

In total, we caught 112 round gobies (TL=9.71 cm±1.54 s.d., WW =12.95 g±6.11 s.d., HW=1.93±0.36 s.d., BD=1.83±0.30 s.d., FR=5.18±0.21 s.d., 60 males, 37 females and 15 juveniles) (Table 1; Table S3) in the harbour Kleinhüningen. The sample contained more male fish than females, and the gender ratio of the total population (male to a female) was 1.62:1. The 15 juvenile individuals (genital papilla were too small to identify gender) were excluded for the gender ratio, size, and mass analysis. The Shapiro–Wilk test indicated normal distribution (*P*<0.05) of the fineness-ratio (FR) among our round goby sample. The shape characteristics of the 3D-printed fish and the rubber ball are provided in Table S4.

**Table 1.**
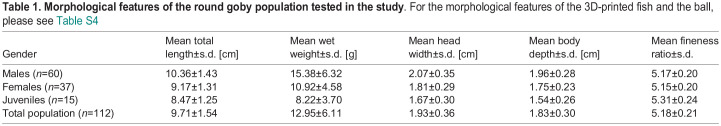
**Morphological features of the round goby population tested in the study**. For the morphological features of the 3D-printed fish and the ball, please see Table S4

### Hydraulic forces experienced by preserved fish, 3D-printed fish and the rubber ball

The preserved fish showed small differences when positioned in the water column or just above the bottom; however, the bottom surface structure provoked significant differences on the mean hydraulic forces when fish were positioned in the water column (Fig. 6; Table S5). In comparison, in the mean hydraulic forces experienced by the rubber ball, the distance to the bottom made a remarkably larger difference. In case of the fish-shaped objects tested (the preserved and 3D-printed fish), the bottom roughness had a larger impact on the mean hydraulic forces experienced than the distance to the bottom (Fig. 6).

When the hydraulic forces experienced by the preserved fish were averaged over the measurement period of 60 s, round gobies experienced significantly different mean hydraulic forces between both bottom roughness treatments in the water column (Column Rough, CR and Column Smooth, CS; adjusted p=0.001, p.adjustment method=“Bonferroni”). Contrary, the mean hydraulic forces remained similar between both bottom roughnesses when measured at the bottom (Fig. 6). In addition, no significant differences in the mean hydraulic forces were detected when the position in the water column was varied. The Kruskal–Wallis test for all treatment combinations indicated significant differences among treatments (*P*-value=0.003; effect size=0.03). Differences between the individual measurements in all experimental regimes were much smaller when preserved fish were studied than when 3D-printed fish were evaluated (Fig. 6).

Overall, the preserved fish experienced mean hydraulic forces of 0.077 N±0.025 s.d. at CR, 0.070 N±0.020 s.d. at bottom rough (BR), 0.070 N±0.020 s.d. at bottom smooth (BS), and 0.068 N±0.021 s.d. at CS.

The largest mean hydraulic forces of 3D-printed fish were recorded with 0.112 N±0.043 s.d. at BR, with 0.105 N±0.050s.d. at BS, with 0.100 N±0.037 s.d. at CR, and the lowest forces with 0.092 N±0.037 s.d. at CS. Reference measurements (hydraulic force measured without fish connected to the fixation stick) revealed the lowest hydraulic forces measured across all treatments and controls tested. Mean hydraulic forces of 0.034 N±0.005 s.d. were measured for BS, 0.035 N±0.003 s.d. for CS, 0.038 N±0.008 s.d. for BR, and 0.041 N±0.011 s.d. for CR were measured for the reference.

The ball displayed the largest mean hydraulic forces measured during the whole experiment with 0.194 N±0.017 s.d. highest at BR. 0.178 N±0.024 s.d. were measured at BS, 0.154 N±0.010 s.d. were measured at CR, and the lowest hydraulic forces of 0.143 N±0.012 s.d. at CS.

The hydraulic forces experienced by the ball, the 3D-printed fish and the reference generally followed the same pattern across the treatments, with larger hydraulic forces experienced at the rough bottom and the lowest forces experienced at the smooth bottom. This pattern was not observed for the preserved fish, as BR and BS experienced similar hydraulic forces.

Fin spread category (class 2: *P*=9,67*10^−13^, class 3: *P*=9,92*10^−8^, class 4: *P*=0.0227), body arching (*P*=0.0035), sex (class 2: *P*=0.0052, class 3: *P*=0.7228), and wet weight (*P*=0.032) were significant shape predictors for the hydraulic forces experienced by the individual fish, as revealed by the generalized linear model. Considering that the live fish can adapt the fin spreading and body arching features while swimming, this result illustrates how the round goby can affect the hydraulic forces experienced in flowing water by adaptation of the fin postures and body arching. The total length, head width, and body depth were no significant predictors of the hydraulic forces experienced by the fish.

### CNN prediction of the treatments on the hydraulic forces time series

The preserved fish experienced characteristic hydraulic forces at the CS treatment, as indicated by a high mean F1 score (F1=0.88±0.03s.d.) (Fig. 7, see Table S5 for the code of data preparation and training the CNN). Because the model was trained on the hydraulic force time series data, we conclude that characteristic patterns within the time series allowed for precise prediction of the CS treatment time series. The mean F1 scores of the BS treatment prediction (F1=0.68±0.05 s.d.) was not as high as the F1 score of CS, but remarkably higher than the mean F1 scores of CR (F1=0.49±0.09 s.d.) and BR (F1=0.43±0.08 s.d.). As indicated by the low F1 scores of CR and BR, we conclude the model did not identify characteristic patterns and was therefore not able to perform reliable prediction of these treatments, namely differences between positioning in the column or at the bottom when the bottom surface was rough from the hydraulic force time series data.

The power spectrum density analysis revealed overall similar power distribution across the treatments. At least four of the highest peaks occurred at the lower frequencies under 66.41 Hz across all treatments. Only BS did not show the peak at 3.91 Hz, but additional peaks compared to the other treatments at 66.41 Hz and 85.94 Hz. It is possible that this deviation from the other treatments was recognized by the CNN and allowed for the highest F1 score observed for BS (see Fig S1 and Table S6).

## DISCUSSION

### The hydraulic forces experienced near the bottom: filling the research gap

We explored whether the round goby has a hydraulic advantage from being near the bottom compared to staying 10 cm above in a flow channel. Our data showed no significant differences in the hydraulic forces between these two positions in preserved and 3D-printed fish. However, with the fin-spread angle and body arching being significant predictors of the hydraulic forces experienced by the fish, we conclude that body posture has a higher relevance for fish to reduce the hydraulic forces in flowing water than their vertical position in the water column. Considering that round goby has a general high affinity to swimming near the bottom (Gilbert et al., 2016), we assume there are further factors causing the fish to swim at the bottom.

In general, the preserved fish bodies were well adapted to equalizing hydraulic forces in flowing water, as the remarkable differences in the hydraulic forces among treatments of the ball, and the less pronounced differences of the 3D-printed fish, were not observed at the preserved fish. The hydraulic forces of the preserved fish were similar among treatments especially between both bottom treatments (BR and BS). But why did the hydraulic forces experienced by the preserved fish differ significantly between the smooth and the rough bottom surface when positioned 10 cm above in the water column? Potentially, the rough gravel surface induced less uniform flow in the water column, leading to increased hydraulic forces experienced by the fish. Because our experiment focused on the hydraulic forces and did not account for flow measurements, we cannot derive conclusions about how the hydraulics affected the forces in detail. ADV measurements of water velocity profiles of the flow channel used in a previous study showed the water velocity was relatively evenly distributed across the measurement chamber (Wiegleb et al., 2020). An idea about how the hydraulic forces depend on the present water velocity is provided by Castagna et al. (2023), who performed a similar experiment and measured the hydraulic force and the flow upstream and downstream a rigid 3D-printed fish model. They found a correlation between water velocity and drag force, as well as correlation between standard deviation and drag force standard deviation.

Contrary to the preserved fish, stronger hydraulic forces were measured for the ball near the bottom compared to the water column. These differences between the hydraulic forces experienced by the preserved fish and the ball may be explained by their individual shapes. The preserved fish and the ball differed remarkably in the higher fineness-ratio (round goby mean fineness-ratio 5.18±0.21 and ball fineness-ratio of 1.00), which illustrates how hydraulically optimized the fish body shape is compared to the ball (Wiegleb et al., 2020; Pennuto and Rupprecht, 2016; Ohlberger et al., 2006), and may explain the different observations of the hydraulic forces among the treatments between the fish and the ball. The 3D-printed fish represented fish-shaped objects with higher rigidity and displayed intermediate hydraulic forces between the forces experienced by the preserved fish and the ball. In addition, the different surface characteristics, such as the skin of the preserved fish in contrast to the plastic surface of the printed fish, as well as the spread fins of one of the fish might have contributed to the discrepancy between the preserved fish and the ball.

Our observations illustrate how well fish are adapted for experiencing comparable low hydraulic forces in flowing water and substrate changes have minor impact on the hydraulic forces experienced by the fish near the bottom. That fish with fusiform shape do likely not experience smaller hydraulic forces when swimming near hard surfaces in flowing water than rough surface agrees with the observation of Downie and Kieffer (2017), who did neither observe a preference for smooth or pebble substrate of *Acipenser brevirostrum*, nor an impact of the bottom substrate on the swimming performance*.* Additionally, Webb (2002) did not observe any effect of the ground distance in the tailbeat frequency and amplitude of swimming cod. An open question remains as to why benthic fish nevertheless prefer swimming near the bottom as reported by Carlson and Lauder (2010), Egger et al. (2021), or Wiegleb et al. (2022). Beside further biological reasons, such as sheltering from predators, another explanation could be the specialized station-holding swimming style (Carlson and Lauder, 2010; Gilbert et al., 2016). Some benthic fish can modify the hydraulic forces in a way that supports station holding by pressing the fish to the bottom without further effort from the fish (Carlson and Lauder, 2010; 2011; Wiegleb et al., 2020). Because this swimming style has such a high relevance for swimming in some benthic fish, it is likely that the fish profit from the opportunity to quickly hold the station and recover when swimming near the bottom against the flow. Switching from burst to station holding would be more difficult in the water column since the fish must first find a location with suitable flow conditions. Another potentially important mechanism supporting station holding in swimming fish has been mentioned by Windsor and McHenry (2009). The water flow around a swimming fish might be altered by its own body and fin movements, which induces signals in the lateral line system in the fish that interfere with signals from the flow that provide information about potential obstacles in the swimming route of the fish. In terms of sensing the environment, station holding might provide a more accurate impression of the surroundings than swimming.

### Is there a hydraulic advantage from staying at the bottom at increased water flow?

A bottom-dwelling fish that swims upstream needs to detach from the substrate to reduce friction with the bottom and produce thrust (Sfakiotakis et al., 1999). Hoover et al. (2003) reported that round gobies preferred to stay on or near the bottom of substrates and spent little time (<20%) swimming in the water column. Pennuto and Rupprecht (2016) reported that round gobies limited their swimming mode to caudal thrusts, swam less frequently, and sought shelter more often as velocity increased. In such a situation when the fish entered the flow field to swim upstream, the round goby was observed switching to a very similar (more subcarangiform) swimming style (Wiegleb et al., 2022) together with surprisingly strong swimming capabilities (Wiegleb et al., 2023b). A comparable swimming behaviour was also observed in the European glass eel, which increasingly showed burst-and-coast swimming at near bottom water velocities exceeding 0.2 m/s (Vezza et al., 2020). The glass eel can make progress upstream only during the burst phase of this swimming style. However, ground effects were observed to not necessarily enhance the performance of undulating fins (Blevins and Lauder, 2013), which agrees with the observations in our study. A hydraulic advantage from swimming at the bottom was only observed in hydraulic force measurements on flapping flexible panels, when the produced thrust was higher near the substrate during the movement amplitudes (Quinn et al., 2014). This observation might explain why we did not observe a significant effect of the fish position in the water column on the hydraulic forces experienced. Our fish were tested in a static position and our measurement did not account for body movements of the fish. We conclude that fish swimming at the bottom do not have a hydraulic advantage *per se* but need to carry out appropriate body and fin movements. Whether a fish glides motionless in the water column or stays near the ground does not seem to have an important effect on the hydraulic forces experienced.

### Hydraulic force time series classification using CNN

CNNs are useful in fish ecology research, e.g. for classification of time series data because they can discover and extract internal patterns in the data series to generate the deep features used for classification (Zhao et al., 2017). This ability allowed to elucidate the hydraulics fish encounter in flowing water, which are complex and highly depend on the shape of the tested fish. Slight variation of the fish body shape can have strong effect on the hydraulic forces experienced, as shown by the result of the linear mixed-effects model, and have the potential to be more pronounced in the data, compared to potentially slight patterns in the time series induced by the bottom substrate. Another advantage of the CNN was its ability to include the time series for the classification task, as comparing the mean hydraulic force among treatments does not account for the temporal proceeding and fluctuation of the hydraulic forces. In addition, the CNN was theoretically able to account for patterns among the three axial channels (X, Y, and Z) because all three channels were provided for training. Thereby, the model could have used these channels to account for the orientation of the overall force vector over the measurement period. Since these patterns are processed internally, we are not able to reveal which patterns contributed to successful classification (Zhao et al., 2017). What we observed was a good model performance when predicting CS, a weaker model performance when predicting BS, and a weak model performance when predicting the time series of CR and BR. Beside the finding that characteristic patterns were identified in CS, this result indicates that only limited characteristic patterns were present in the time series recorded above the rough bottom, indicating the time series of these treatments proceeded similarly. This observation shows that even bad model performance for a category can serve as a meaningful result. Because the model performed quite well for predicting CS, we conclude that the model was generally able to identify and use patterns within the data for classification.

### Relevance for the round goby invasion

The majority of the world's largest river systems are fragmented (Nilsson et al., 2005) and the restoration of degraded ecosystems is requested, e.g. by the Convention on Biological Diversity. Research mainly focuses on swimming of economical relevant species (Aarestrup et al., 2003; Jansen et al., 1999). With the round goby, a benthic fish species, it became the focus because its expansion represented a threat to the local biodiversity and local fisheries, and because it serves as model species for other benthic small fish (Kornis et al., 2012; Adrian-Kalchhauser et al., 2020; Cerwenka et al., 2023). Several similarities in the swimming style have been reported with bullhead, which exhibits similar station-holding behaviour to the round goby (Egger et al., 2021; Wiegleb et al., 2022). Nevertheless, there were also differences in the hydraulic forces observed among species, as gudgeon experienced significantly lower hydraulic forces in a vertical-slot fish pass and over the hydraulic barrier than round goby (Wiegleb et al., 2022; 2023a). This observation indicates that other species might experience different hydraulic forces under the conditions of the present study and shows the fish species react differently to the hydraulic forces they experience. Fish swimming behaviour is complex and depends on numerous factors, such as activity, exploration, and boldness (Cano-Barbacil et al., 2020; Lothian and Lucas, 2021). Fish species diverge not only in their body shape and swimming style, but also in the hydraulic forces they are exposed to and that they themselves induce in the water flow. Since hydraulic forces determined by river morphology and the design of fish passes have a direct impact on the fish, hydraulic forces represent a direct measure of the physical impact of restoration measures on the fish. With our study, we provide one step towards understanding how the ground structure affects benthic fish physically while swimming.

### Further questions to be answered

The experiment described was a standardized assessment of the hydraulic forces static fish bodies experience at the four scenarios tested. With respect to the special case of the round goby, the tested water velocity of 0.95 m/s was above U_crit_ value measured for round goby [54.0 cm/s, Egger et al. (2021); 35.5 cm/s Tierney et al. (2011)] but under the maximum burst speed of round goby in still water (136 cm/s) (Tierney et al., 2011). To further explore the hydraulic forces from the water flow experienced by fish near the bottom, we recommend testing further water-flow velocities, bottom structures, fish sizes, and fish species.

In a scenario where a fish attempts to pass an area of increased flow, its swimming behaviour is likely affected by the turbulence encountered (Silva et al., 2012). Vortices of specific size have the potential to destabilize a swimming fish (Tritico and Cotel, 2010), therefore a next-step study should account for the body movements of swimming fish, as well as the water flow around the swimming fish. For instance, Adhikari and Longmire (2013) were able to measure the water flow around foraging zebrafish using infrared particle image velocimetry. Together with a state-of-the-art tracking tool, such as DeepLabCut (Lauer et al., 2022), it might be feasible to describe the effect of flow conditions on the behaviour of fish swimming near the bottom.

## MATERIALS AND METHODS

### Fish catching

In total, 112 round goby (*N. melanostomus* Pallas, 1814) fish were caught in the harbour Kleinhüningen, Basel, Switzerland in July 2019. We used minnow traps baited with dog food (Frolic^®^). The minnow traps were exposed in the harbour overnight and caught fish were euthanized in a solution of clove oil immediately after catching (Fernandes et al., 2017). The euthanized fish were counted, placed in an ice box, and transported to the lab immediately, where the fish were preserved in a mixture of formalin and ethanol (Wiegleb et al., 2020).

### Fish preservation

Total length (TL) and wet weight (WW) of the fish were measured (Mettler Toledo PL1502-S) and sex was determined visually based on the shape of the genital papilla (Kornis et al., 2012). Afterward, the fish were preserved in formalin (4%; 72 h) with their fins laid onto their body and adjusted to be straight and symmetric using needles. Wiegleb et al. (2020) assume that this body posture best represents a fish swimming posture while swimming against the flow because the body surface area exposed to the flow is minimized when the fins are close to the body. The formalin solution was then stepwise replaced by ethanol (75%) (Wiegleb et al., 2020).

### Morphometric measurements

**Fig. 1.**
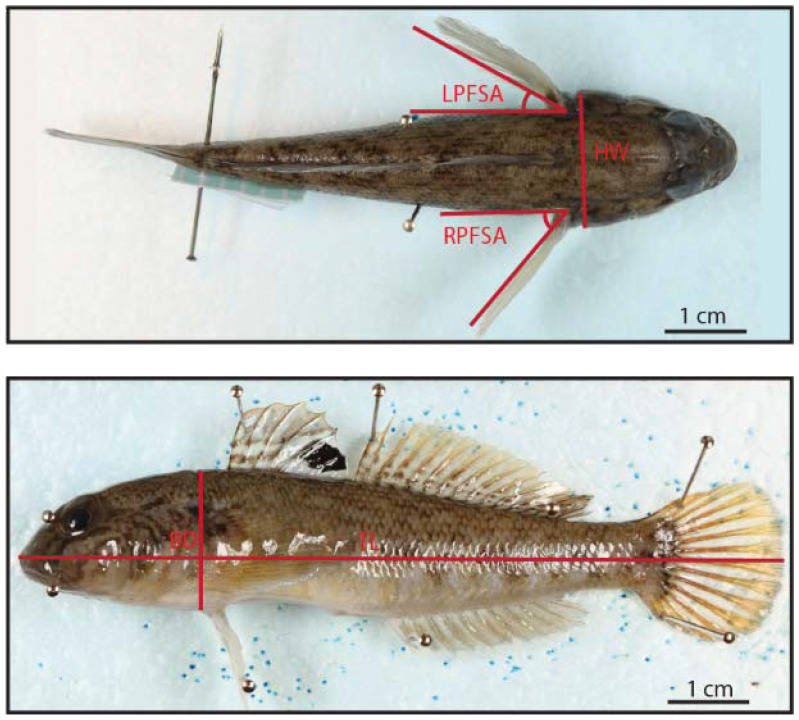
Morphometrics measured for the fish in the dorsal (top) and lateral (bottom) view.

To describe the fish shape and body posture after the preservation, we photographed the fish from lateral, dorsal, and ventral perspectives using a Canon EOS 70D camera and measured the morphometric characteristics of the fish in ImageJ (Version 1.53f51) (Fig. 1). The morphological characteristics included the mean of both pectoral fin-spread angles per fish (MPFSA), which was calculated from the left pectoral fin-spread angle (LPFSA) and the right pectoral fin-spread angle (RPFSA), then fin spread was categorized between 1 and 4 (non-spread, slightly spread, medium spread and larger spread, based on the MPFSA variance). Although most fish maintained their body straightness after the preservation, some bodies bent slightly. The fish body arching angle was quantified and categorized between 1 and 4 (straight, slightly bent, medium bent, and larger bent based on the MPFSA body arching angle variance). The fish fin spread and body bent were measured to assess the influence of body shape on hydraulic forces.

We further computed the body FR to assess the influence of body shape on hydraulic forces. Engineers and biologists use the FR of an elliptical body of revolution, the ratio of major to the minor body axis, to determine the influence of shape on the drag of a moving body in water (Landweber, 1961; Webb, 1975; Blake, 1983). Each individual's FR was calculated with the T_L_, the maximum head width (HW), and maximum body depth (BD) (Ohlberger et al., 2006).

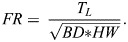


To increase the comparability of our study, we computed the Reynolds number (Re):

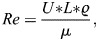
with U being the water velocity (0.95 m/s), L being the mean length of preserved fish (0.097 m), 3D-printed fish (0.076, 0.073, and 0.093 m), or diameter of the rubber ball (0.03 m), ϱ being the density of water (998.2 Kg/m^3^) and *μ* being the dynamic viscosity of water (0.001308 pa*s). The Reynolds number for the preserved fish was 0.70*10^−5^; for 3D_Fish_1 0.55*10^−5^; for 3D_Fish_1 0.53*10^−5^; for 3D_Fish_1 0.67*10^−5^; and for the rubber ball 0.22*10^−5^. The original images of the preserved fish and the 3D-printed fish are available in the Figshare repository (please see the data and resource availability statement).

### Controls

Although the body shape of preserved fish is very close to the shape of live fish, it is likely that a live fish will behave differently and experience similar but different hydraulic forces in flowing water. To provide an idea of how material and shape affect the hydraulic forces observed, we included two control treatments in our study. Beside the preserved fish, we measured the hydraulic forces for a more rigid fish body, a 3D-printed round goby (*n*=3) and a simple spheric shape (a rubber ball, material: acrylonitrile-butandiene, diameter: 3.00 cm). The 3D-printed fish were similar in shape to the preserved fish but consisted of a different material and had a different surface structure. The rubber ball provided a simple shape that was far removed from the hydraulically optimised shape of a fish. The preserved round goby were scanned using a Shiny 3D scanner^®^ and the 3D model obtained was prepared for 3D printing in Blender v.2.79. The optimized model was then printed using a Formlabs Form 2^®^ printer with Formlabs Photopolymer Resin Clear (FLPCL04), cleaned in isopropanol, and cured under UV light for 20 min. The hydraulic forces experienced by the 3D-printed fish and the spheric rubber ball were then measured for the same treatments and in the same manner as the preserved round goby, and the shape characteristics of the 3D-printed fish and the ball were measured the same as the preserved fish.

### Force measurement

**Fig. 2.**
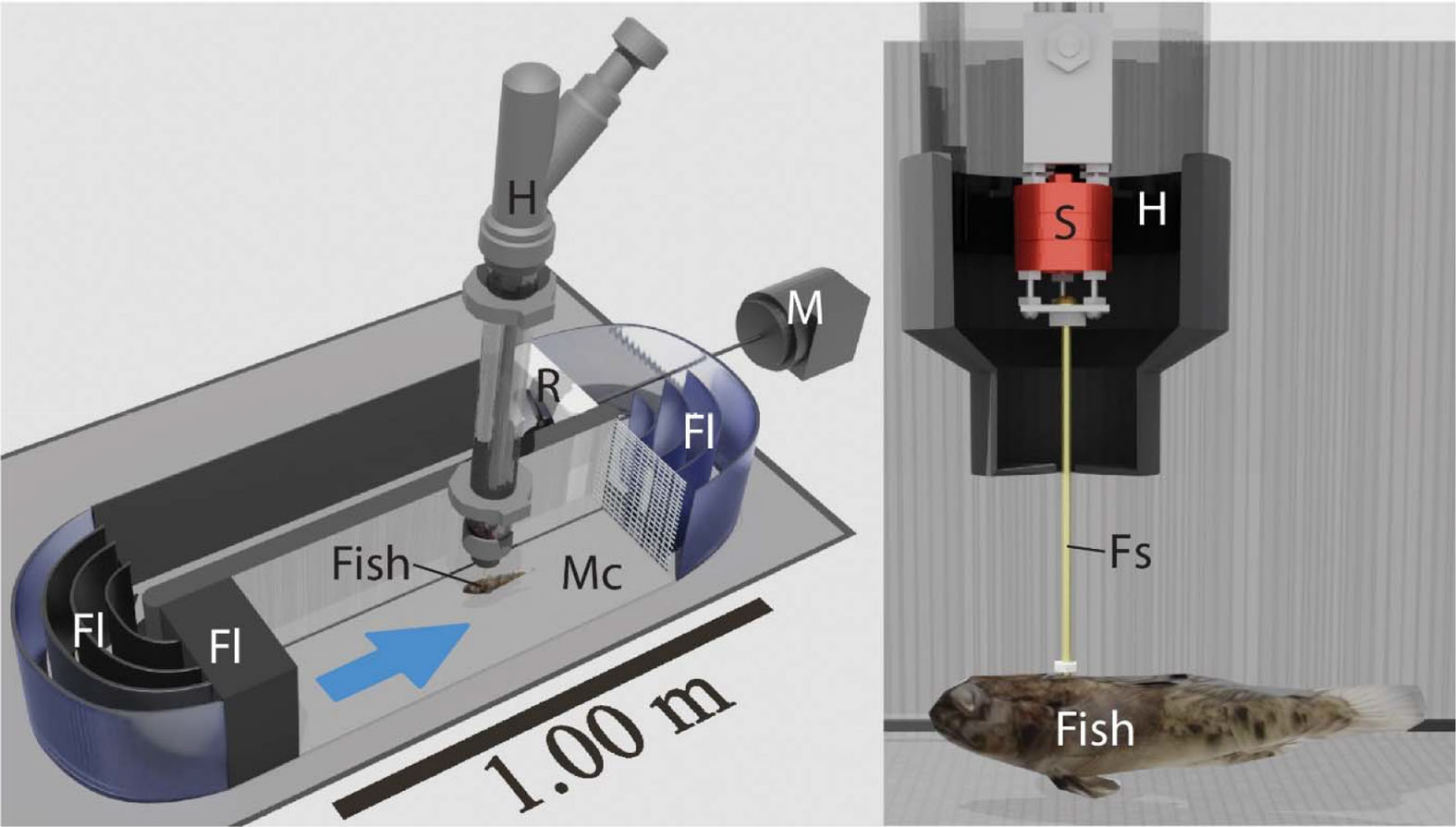
**Flow channel with the measurement probe.** The circular water flow was driven using an electric engine (M), which transduced the moment to a rotor (R) via a drive shaft. The water flow was straightened using different flow straighteners (Fl). The flow direction is indicated by the blue arrow. In the measurement chamber (Mc), the fish was connected to the force sensor (S) via a fixation stick (Fs). The sensor was protected against the lateral flow with a Polyvinyl-chloride-hull (H).

**Fig. 3.**
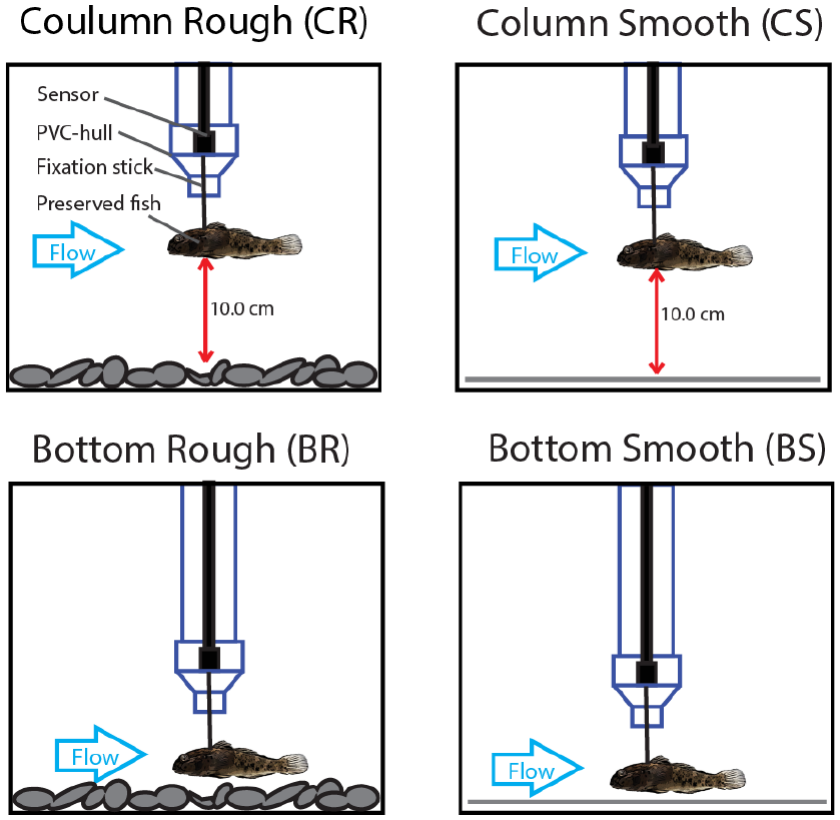
**The hydraulic forces were measured for four treatments in the flow channel.** Preserved fish were measured 10 cm above the bottom at the gravel bottom substrate (CR) and smooth plexiglass substrate (CS) and directly above the bottom (distance to the bottom: 5 mm) at gravel substrate (BR) and smooth plexiglass substrate (BS). It was ensured the fish had no physical contact with the bottom and gravel to avoid uncontrolled friction forces affecting the force measurements.

The hydraulic forces experienced by the fish were measured in the standardized flow of a Loligo Systems^®^ (185 L, 50 Hz, Tjele, Denmark) flow channel (Fig. 2). To test whether the fish experience lower hydraulic burden at the bottom, we measured the hydraulic force experienced by the fish near the smooth bottom (5 mm above to avoid physical contact between fish and bottom) of the flow channel (BS) and in the water column, 10 cm above the smooth bottom (CS) (Fig. 3). To test how the bottom substrate may affect the hydraulic forces experienced, we performed the same experiment with an external bottom inlet with gravel particles glued on its surface. We determined the mean size (Ø=2.48 mm±0.73 s.d.) of the gravel particles using ImageJ from a sample of 100 particles. Hydraulic forces were measured directly above the rough bottom (BR) and in the water column, 10 cm above the gravel (CR). We performed the experiment in the same flow channel applied by Egger et al. (2021) and applied the same 3D-force measurement probe applied by Wiegleb et al. (2022).

The force measurement probe contained a water-resistant high-precision-3D-Force-Sensor (ATI Industrial Automation, F/T Sensor: Nano17 IP68, calibration SI-50-0.5, calibrated by the producer), which was sheltered against the lateral flow with a stable polyvinyl-chloride (PVC) hollow cover. The preserved fish were punctuated in the assumed center of gravity (Sagnes et al., 2000; Wiegleb et al., 2020) and connected to the force sensor via a 10 cm long (3 mm diameter) on the brass stick with one dorsal and one ventral nut. This fixation stick acted as a lever, which transduced the force acting on the fish to the sensor, similar to Wiegleb (2022) and Wiegleb et al. (2020). The fish were always oriented with their head against the water flow.

After fixing the preserved fish at the fixation stick, the probe was placed in the centre of the flow channel measurement chamber. The probe was adjustable in height to position the fish for the treatment to be tested. The force sensor was reset before increasing the water discharge to a water velocity of 0.95 m*s^−1^. This water velocity was chosen because it was below the velocity range that induced vibration in the force measurement system (Wiegleb et al., 2020). When the desired water velocity was achieved, we started the data recording of the sensor manually and recorded the torque in the X-and Y-direction and the force in the Z-direction with a sampling rate of 1000 Hz and recorded for 60 s. The forces were measured for every fish and at every treatment once. The hydraulic forces were recorded with a mean signal-to-noise ratio of 12.10±4.59 s.d. for CR 13.69±4.48 s.d. for CS, 13.30±4.26 s.d. for BR, and 13.13±3.92 s.d. for BS for the preserved fish.

After the experiment, we computed the hydraulic force experienced by the fish in X- and Y-direction from the measured torques in X-and Y-direction using the moment arm formula (Davidovits, 2024; Tipler and Mosca, 2003) in MATLAB (R2019a):

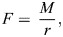
with F being the force [N], M being the torque [Nm], and r being the length of the lever brass stick [m], which was 0.10 m. With the mean hydraulic forces of each dimension (X-, Y-, Z-direction), we computed the strength of the mean 3D hydraulic force [N] (F_3D_) experienced over the measurement period:

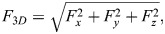
with F_X_ being the mean hydraulic force in X-direction, F_Y_ being the mean hydraulic force in Y-direction, and F_Z_ being the mean hydraulic force in Z-direction. A fixation stick ensured the fish were exposed to the flow at the desired position in the flow channel. To describe the effect of this fixation stick in the forces measured, we performed a reference measurement without fish connected to the fixation stick (four measurements for every treatment).

### Statistics

We performed all statistical analyses in R v.4.0.4 and RStudio v.1.4.1106. Significant differences in the mean hydraulic forces between treatments were assumed when *P*<0.05. We tested the data for normal distribution using the Shapiro–Wilk test. Because the force data were not distributed normally, we then applied a Kruskal–Wallis test to test for differences in the means between treatments and computed the effect size (Tomczak and Tomczak, 2014).

Afterward, we performed pairwise comparisons between groups using Dunn's test with the conservative Bonferroni correction method (Dunn, 1961). The Bonferroni correction method was necessary to control the Family-Wise Error Rate due to multiple comparisons between the treatments.

To detect the morphological variables with the largest impact on the hydraulic forces experienced by the preserved fish, we performed a Generalized Linear Model (glm() function)(Nelder and Wedderburn, 1972). The explanatory variables were fish gender (Sex) with ‘male’, ‘female’, and ‘undefined’ as categories, the wet weight (WW) in g, total length (TL) in cm, head width (HW) in cm, body depth (BD) in cm, fin spread (FinSpread) with ‘straight’, ‘slightly bent’, ‘medium bent’, and ‘larger bent’ as categories, and body arch (Arch) in degrees. ‘ID’ represents the identification number of every fish.

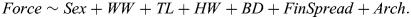


The generalized linear model based on inverse Gaussian family “log” link function was chosen to handle the left-skewed and non-normally distributed force data. With the chosen configuration, the model showed a large reduction of the null deviance (511.54) to the residual deviance (183.92), indicating the model explained a substantial amount of variance in the hydraulic force.

### Using Artificial Intelligence to predict the treatments on the hydraulic forces

Besides the comparison of the mean hydraulic forces, we compared the fluctuations of the hydraulic forces over time. To account for complex patterns in the time series force data, such as various amplitudes and frequencies of the three hydraulic force channels (X-, Y-, and Z-directed force), we trained a deep CNN on the time series of the force data to predict the treatments based on patterns within the data. If the CNN achieved a high prediction accuracy for a treatment, we concluded that the time series exhibited characteristic patterns that allowed the model for categorization. Training CNNs requires large data amounts (Zhao et al., 2017). If trained on sufficient data, the model identifies patterns used for categorization itself, which makes this approach useful for complex time series (Zhao et al., 2017), such as our data.

A power spectrum density analysis of hydraulic forces experienced by the preserved fish was performed for the different treatments to visualize patterns in the data that were potentially used by the CNN for classification (see Table S1 for the code). A median power spectrum density curve was computed across all fish of a treatment and the frequencies at the five curve peaks were determined for comparisons across treatments.

### Data preparation

**Fig. 4.**
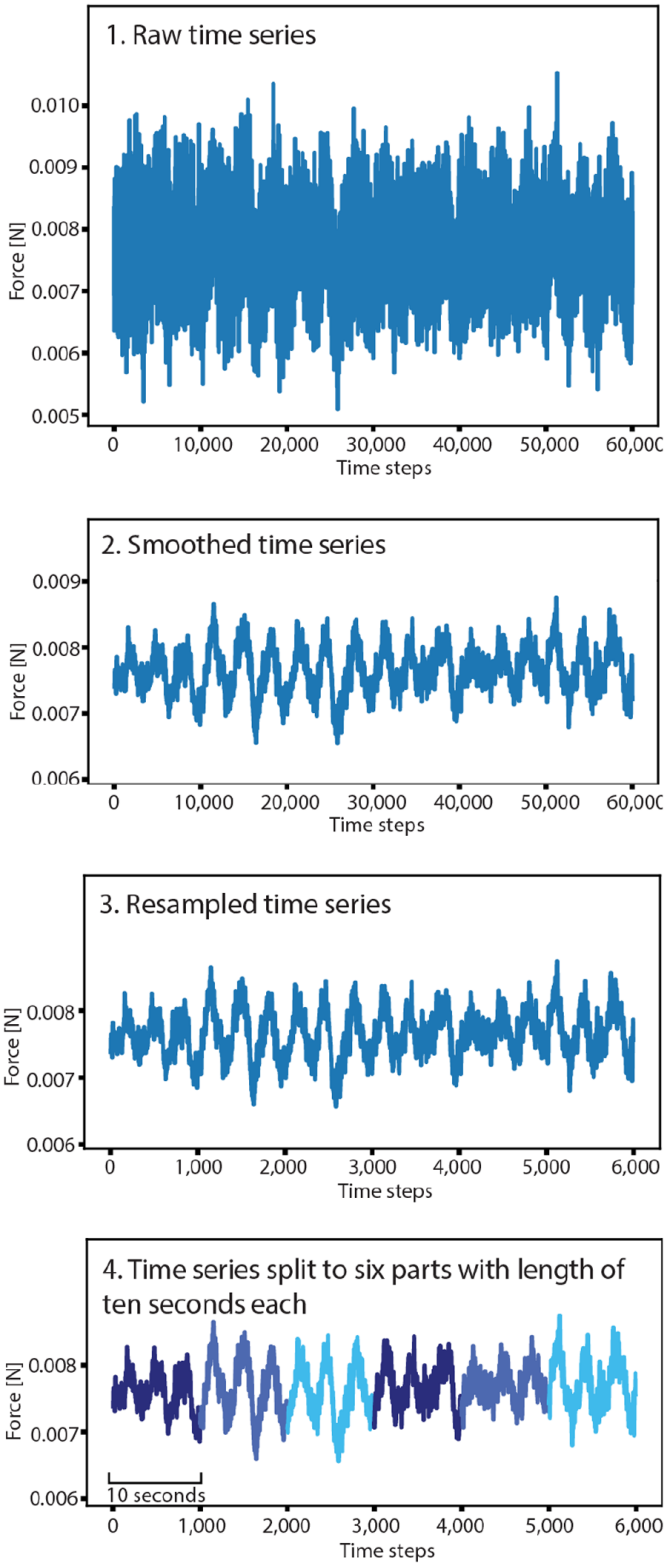
**preparation of the time series data for training the neural network.** The raw times series (1) was smoothed to reduce noise (2). Then, the data were resampled to reduce the computational effort when training the neural network (3). Because neural networks require large amounts of training data, the time series was split into six parts with a length of 10 s each (4).

The raw time series data were prepared for cross-validation of the CNN (Fig. 4). The X-, Y-, and Z-time series of the force measurements were smoothed slightly. To reduce the information fed to the CNN, we downsampled the time series to 10% from the total time steps (resulting in 6000 observations). To increase the number of time series and to obtain sufficient data amounts for reliable prediction, we split every time series into six parts of equal size (length of 1000 observations each with a duration of 10 s). Data sets of the corresponding labels were created with values of 0 for ‘CS’, 1 for ‘CR’, 2 for ‘BR’, and 3 for ‘BS’. The data were split into three groups of the same size for cross-validation. To increase the data set further, we repeated the entire procedure twice with different smoothing adjustments. We thereby obtained three data sets with slightly different time series but with an overall similar appearance. This simple method of data augmentation (Taylor and Nitschke, 2018) was used to increase the amount of training data and thereby improve the model accuracy. Finally, we combined the data sets of every smoothing kernel to obtain three data sets with an equal proportion of every smoothing kernel used and an equal proportion of every treatment to obtain balanced cross-validation data sets. Two data sets were then combined into a training data set (5376 time series) and one validation data set (2688 time series), whereby the validation data set contained time series not contained in the training sets – also not with a different smoothing kernel. Thereby, we ensured the model was validated with completely unknown time series, which were not included in the training process. The cross-validation was performed for different training and validation data set combinations to validate the CNN on all force time series recorded in the experiment. We computed the model accuracy Ac_a_ for treatment as follows:

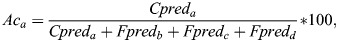
with Cpred_a_ being the number of correct predictions, Fpred_b_ being the number of false predictions to be ‘a’, although it is treatment ‘b’, Fpred_c_ being the number of false predictions to treatment ‘a’ although it is treatment ‘c’, and Fpred_d_ being the number of false predictions to treatment ‘a’ although it is treatment ‘d’.

### The CNN model architecture

**Fig. 5.**
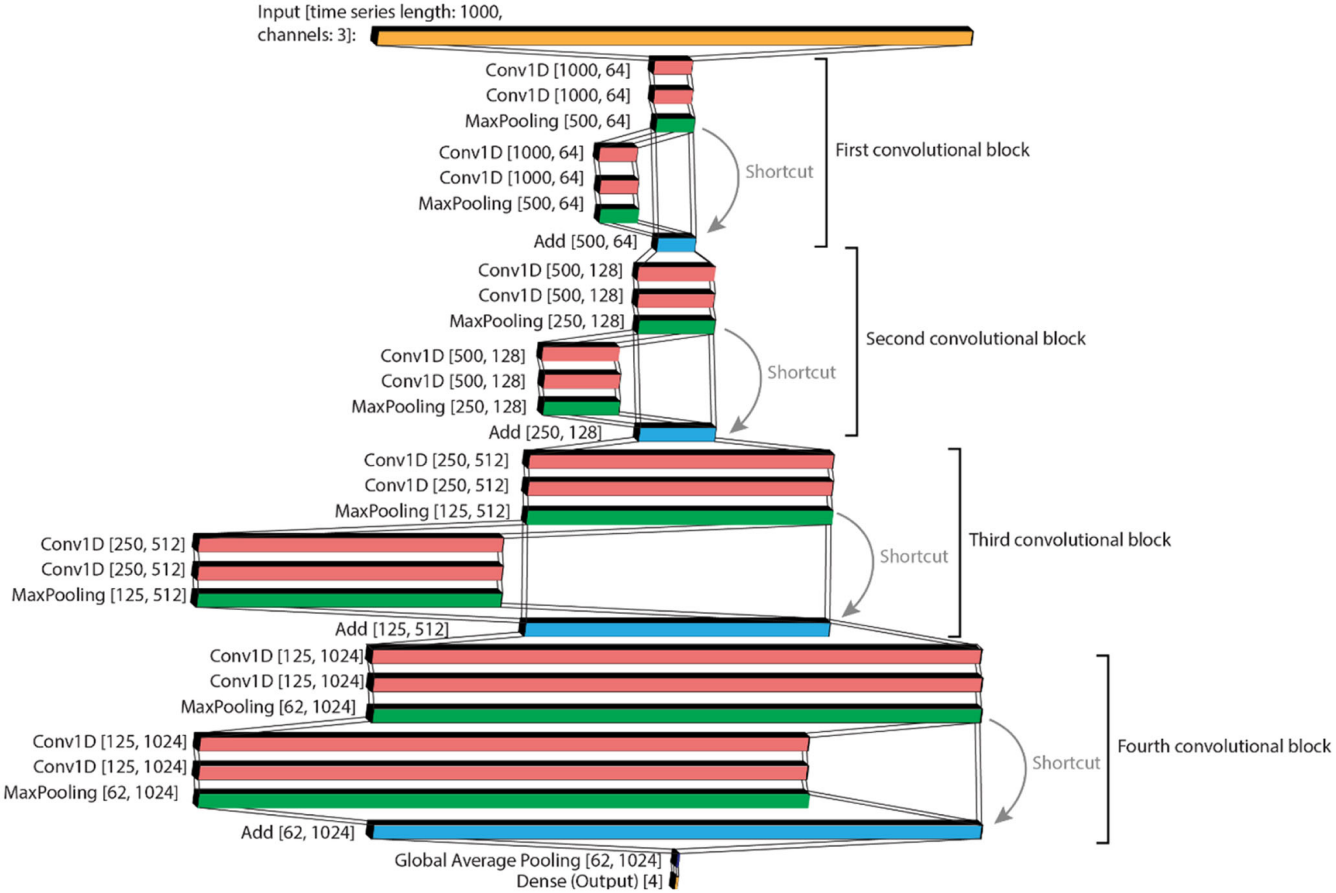
**We created a CNN consisting of four convolutional blocks with increasing numbers of filters.** Shortcuts were implemented, which allowed for the construction of deep networks that was able to identify patterns of high complexity. The MaxPooling layers reduced the length of the time series and thereby reduced overfitting. The Global Average Pooling layer condenses the information at the end of the network and releases the information directly to the output layer.

We created a deep CNN in Keras for the categorization of the time series of the hydraulic forces to the treatments (Fig. 5). If the CNN was able to discriminate between treatments with high accuracy, we concluded the model identified patterns within the time series that allowed for discrimination. CNNs are inspired by the structure of the visual cortex of animals and can recognize complex patterns from data on their own with minimal data pre-processing, such as raw pixels (Gu et al., 2018). We used a simple CNN network architecture (Fig. 5), consisting of four convolutional blocks. Every convolutional layer (Conv1D) provides filters (convolutions) that can be activated with the common ‘relu’ activation function if specific patterns are identified in the trajectory. More complex patterns lead to activations at higher levels. The number of filters is increased with the level of the convolutional block, which increases the number of patterns the model can recognize in the time series. To account for the accuracy degradation problem of deep CNNs, we implemented shortcuts (He et al., 2016). At the end of the network, the information is condensed in the ‘Global Average Pooling’ layer, which feeds the neurons directly to the ‘Dense’ output layer. This output layer is activated with a ‘softmax’ activation function and provides four outputs, one for each prediction category. We implemented MaxPooling layers at the end of every convolutional block and a Dropout layer in the fully connected part of the model to prevent the model from overfitting while training. Please see Gu et al. (2018) for further details about the design of CNNs. The model provided a total of 14,250,244 trainable parameters and was trained for 100 epochs. The code for the CNN analysis, as well as the power spectrum density analysis, was created with support of ChatGPT (GPT-4 architecture).

**Fig. 6.**
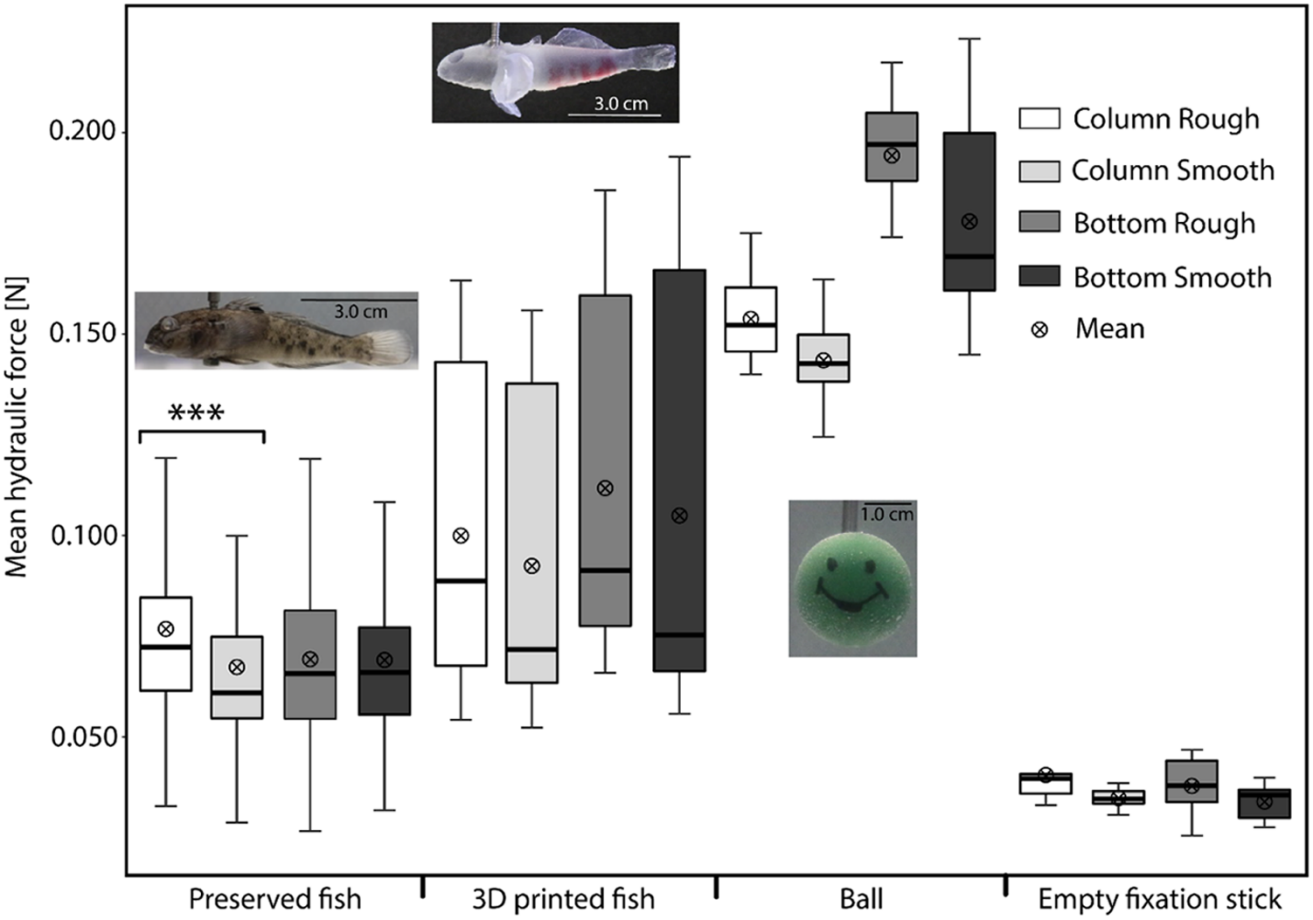
**The mean hydraulic forces of the preserved round gobies (preserved fish, *n*=112), the 3D-printed round gobies (3D-printed fish, *n*=3), the rubber ball (ball, *n*=1), and the empty fixation stick (*n*=1), measured for the different treatments over a period of 1 min.** Significant differences (*P*<0.05) between treatments are marked with asterisks.

**Fig. 7.**
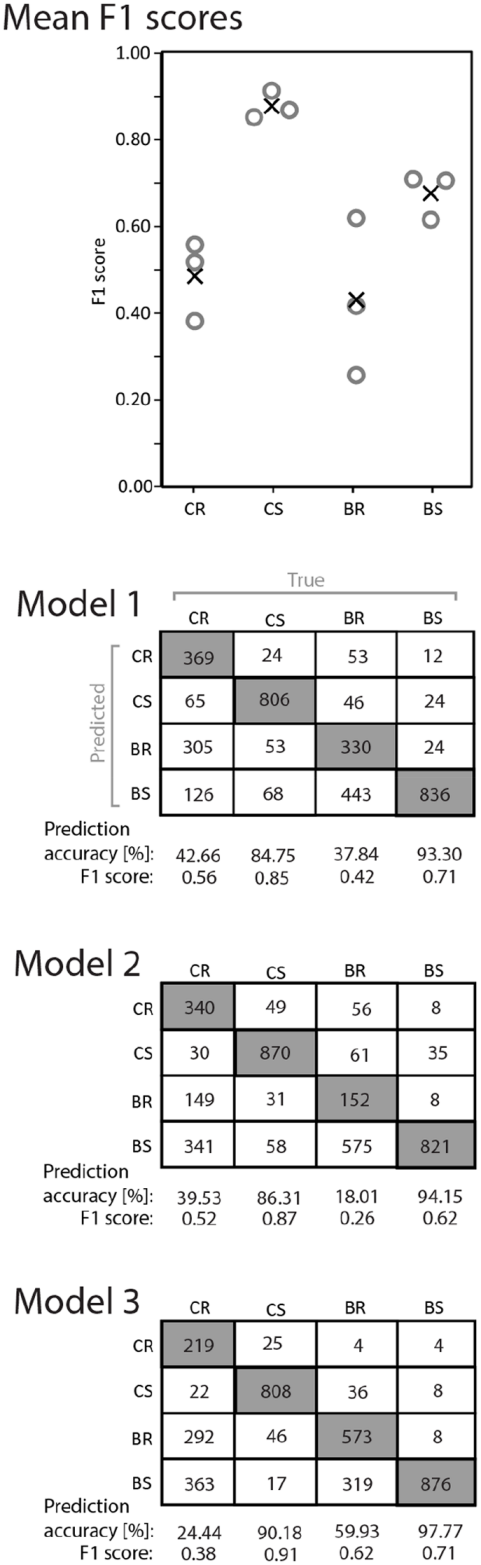
**F1 scores (circles) and mean F1 scores (X) for the treatments.** (CR, Column Rough; CS, Column Smooth; BR, Bottom Rough, BS, Bottom Smooth) of the cross-validation of the CNN (top). An F1 score of 1.0 indicates perfect model performance, whilst an F1 score of 0.0 indicates the worse model performance. Confusion matrices of the three cross-validation models are provided below. Numbers in the confusion matrices represent the numbers of predicted time series for the treatments. For instance, 369 time series were correctly predicted to be CR by model 1. Contrarily, 65 time series were mistakenly predicted as CS, while observed as CR.

## Supplementary Material

10.1242/biolopen.060533_sup1 Supplementary information

Table S2. Fish catchment data

Table S4. Mean hydraulic forces and morphometric data
