# Supplementary material for: Does swimming at the bottom serve as a hydraulic advantage for benthic fish Neogobius melanostomus Pallas (1814) in flowing water?
Source: Biol Open. 2024 Oct 30;13(11):bio060533. doi: 10.1242/bio.060533 (PMC11575849; doi:10.1242/bio.060533)
Supplement: Supplementary information [file biolopen-13-060533-s1.pdf]

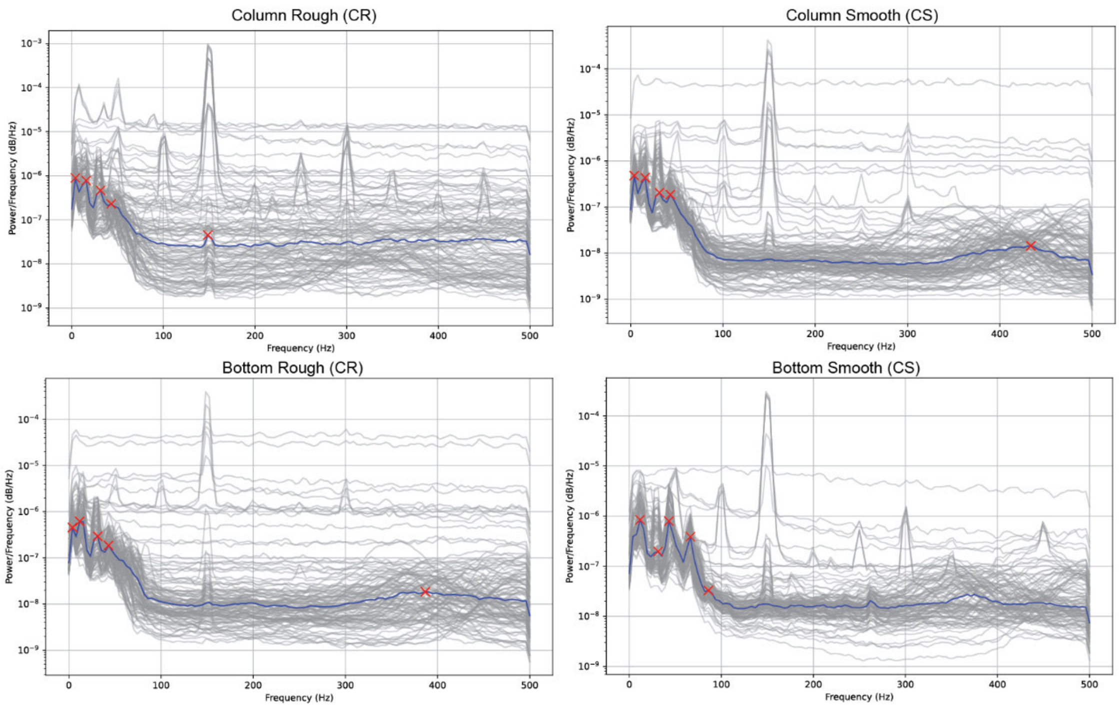

Fig. S1. Median Power Spectrum Density (blue line) for the four treatments. The Power Spectrum Densities of the single fish are represented by the grey curves. The five power maxima of the curves are marked red. The exact values for power and frequency of these maxima are provided in Table S6.

Biology Open • Supplementary information

Table S1. Compute and plot Power Spectral Density from force time series data

```
#written in python
#Author: Joschka Wiegleb


import os
import scipy
import numpy as np
import math

class Maxima:
    def __init__(self):
        self.a_freq = []
        self.a_psd = []
        self.b_freq = []
        self.b_psd = []
        self.c_freq = []
        self.c_psd = []
        self.d_freq = []
        self.d_psd = []
        self.e_freq = []
        self.e_psd = []
        self.treatment = []

def dispMaxima(maxi):
    print(str(maxi.treatment) + "________Point_______freq_______psd")
    print("1.          " + str(maxi.a_freq) + "            " +
str(maxi.a_psd))
    print("2.          " + str(maxi.b_freq) + "            " +
str(maxi.b_psd))
    print("3.          " + str(maxi.c_freq) + "            " +
str(maxi.c_psd))
    print("4.          " + str(maxi.d_freq) + "            " +
str(maxi.d_psd))
    print("5.          " + str(maxi.e_freq) + "            " +
str(maxi.e_psd))

def assignPeaks(freq, mean_psd, treatment, top_peaks):
    Maxi = Maxima()
    Maxi.a_freq = freq[top_peaks][0]
    Maxi.a_psd = mean_psd[top_peaks][0]
    Maxi.b_freq = freq[top_peaks][1]
    Maxi.b_psd = mean_psd[top_peaks][1]
    Maxi.c_freq = freq[top_peaks][2]
    Maxi.c_psd = mean_psd[top_peaks][2]
    Maxi.d_freq = freq[top_peaks][3]
    Maxi.d_psd = mean_psd[top_peaks][3]
    Maxi.e_freq = freq[top_peaks][4]
    Maxi.e_psd = mean_psd[top_peaks][4]
    Maxi.treatment = treatment
    return Maxi


def CompForce(x,y,z):
    force = np.sqrt(x**2 + y**2 + z**2)
    return force

def LoadFiles(path):
```

Biology Open • Supplementary information

```
    data_list = []
    files = os.listdir(inipath)
    for file in files:
        path=os.path.join(inipath,file)
        mat = scipy.io.loadmat(path)
        data = mat.items()
        liste = list(data)
        ty=liste[0][1][5,:]
        tx=liste[0][1][4,:]
        z = liste[0][1][3,:]
        if len(ty)>60000:
            ty=ty[0:60000]
        if len(tx)>60000:
            tx=tx[0:60000]
        if len(z)>60000:
            z=z[0:60000]
        fx = ty/0.1 #fx is computed from ty because ty is the torque
around x axis
        fy = ty/0.1
        force = CompForce(fx,fy,z)
        data_list.append(force)
    return data_list


import numpy as np
import matplotlib.pyplot as plt
from scipy.signal import welch, find_peaks

def PSD(data):
    sampling_rate = 1000  # Sampling rate in Hz
    freq_list = []
    psd_list = []
    peaks_list = []
    for sequence in data:
        # Calculate the Power Spectral Density (PSD) using Welch's method
        frequencies, psd = welch(sequence, fs=sampling_rate, nperseg=256)
        freq_list.append(frequencies)
        psd_list.append(psd)

        # Find peaks in the PSD
        peaks, _ = find_peaks(psd, height=0)  # adjust 'height' to filter
out small peaks
        peaks_list.append(peaks)
        peak_frequencies = frequencies[peaks]
        peak_values = psd[peaks]
    return freq_list, psd_list, peaks_list


import matplotlib.pyplot as plt


def Plot_PSD(freq_list, psd_list, peaks_list):

    plt.figure(figsize=(10, 6))
    for i in range(len(freq_list)):
        plt.semilogy(freq_list[i], psd_list[i], color='grey', alpha=0.4,
label=f'PSD {i+1}')
    plt.title('Power Spectral Density (PSD) with Peaks')
```

```
    plt.xlabel('Frequency (Hz)')
    plt.ylabel('Power/Frequency (dB/Hz)')
    plt.grid(True)
    #plt.legend()
    plt.show()


import matplotlib.pyplot as plt

import matplotlib.pyplot as plt

def Plot_PSD_all(freq_list, psd_list, peaks_list):

    for i in range(0,len(freq_list)):
        plt.figure(figsize=(10, 6))

        # Plot all the PSDs in the background with grey color and alpha 
0.4
        for j in range(0,len(freq_list)):
            plt.semilogy(freq_list[j], psd_list[j], color='grey', 
alpha=0.4)

        # Highlight the current PSD in blue
        plt.semilogy(freq_list[i], psd_list[i], color='blue', alpha=1.0, 
label=f'PSD {i+1}')

        # Update the title with the current iteration number
        plt.title(f'Power Spectral Density (PSD) - Iteration {i+1}')
        plt.xlabel('Frequency (Hz)')
        plt.ylabel('Power/Frequency (dB/Hz)')
        plt.grid(True)
        plt.legend()
        plt.show()




def Plot_Average_PSD(freq_list, psd_list, treatment, save_path, 
confidence=0.95, num_maxima=5):

    # Ensure all PSD arrays have the same frequency bins
    freq = freq_list[0]

    # Stack all PSD arrays to calculate mean and std deviation
    psd_array = np.stack(psd_list, axis=0)

    # Calculate the mean and standard deviation of the PSDs
    mean_psd = np.mean(psd_array, axis=0)
    std_psd = np.std(psd_array, axis=0)

    # Calculate the standard error of the mean
    sem_psd = std_psd / np.sqrt(len(psd_list))

    # Calculate the confidence intervals
    z_score = 1.96  # For 95% confidence interval
    ci_upper = mean_psd + z_score * sem_psd
    ci_lower = mean_psd - z_score * sem_psd
```

Biology Open • Supplementary information

```
    # Ensure that lower CI limits do not fall below a practical limit
    # Find peaks in the mean PSD
    peaks, _ = find_peaks(mean_psd)

    # Sort peaks by their PSD values and select the top 'num_maxima'
    sorted_peaks = sorted(peaks, key=lambda x: mean_psd[x], reverse=True)
    top_peaks = sorted_peaks[:num_maxima]

    maxi = assignPeaks(freq, mean_psd, treatment, top_peaks)

    # Plot the results
    plt.figure(figsize=(10, 6))
    plt.semilogy(freq, mean_psd, color='blue', label='Mean PSD')
    plt.fill_between(freq, ci_lower, ci_upper, color='blue', alpha=0.2,
label='95% Confidence Interval')
    #plt.scatter(freq[top_peaks], mean_psd[top_peaks], color='red',
marker='x', s=100, label=f'Top {num_maxima} Maxima')
    plt.scatter(freq[top_peaks], mean_psd[top_peaks], color='red',
marker='x')

    # Set y-axis limits
    plt.ylim(1e-12, 1e-7)

    plt.title('Average Power Spectral Density (PSD) with 95% Confidence
Interval and Top Maxima for ' + treatment)
    plt.xlabel('Frequency (Hz)')
    plt.ylabel('Power/Frequency (dB/Hz)')
    plt.grid(True)
    plt.legend()
    plt.savefig(save_path, format='pdf')
    #plt.show()
    return ci_lower, ci_upper, maxi

def Plot_PSD_average(freq_list, psd_list, peaks_list, save_path,
num_maxima=5):

    # Ensure all PSD arrays have the same frequency bins
    freq = freq_list[0]

    # Stack all PSD arrays to calculate mean and std deviation
    psd_array = np.stack(psd_list, axis=0)

    # Calculate the mean and standard deviation of the PSDs
    mean_psd = np.median(psd_array, axis=0)
    std_psd = np.std(psd_array, axis=0)

    # Calculate the standard error of the mean
    sem_psd = std_psd / np.sqrt(len(psd_list))

    # Calculate the confidence intervals
    z_score = 1.96  # For 95% confidence interval
    ci_upper = mean_psd + z_score * sem_psd
    ci_lower = mean_psd - z_score * sem_psd

    # Find peaks in the mean PSD
    peaks, _ = find_peaks(mean_psd)

    # Sort peaks by their PSD values and select the top 'num_maxima'
```

```
    sorted_peaks = sorted(peaks, key=lambda x: mean_psd[x], reverse=True)
    top_peaks = sorted_peaks[:num_maxima]

    maxi = assignPeaks(freq, mean_psd, treatment, top_peaks)

    plt.figure(figsize=(10, 6))
    for i in range(len(freq_list)):
        plt.semilogy(freq_list[i], psd_list[i], color='grey', alpha=0.4, 
label=f'PSD {i+1}')

    # Ensure all PSD arrays have the same frequency bins
    freq = freq_list[0]

    # Stack all PSD arrays to calculate mean and std deviation
    psd_array = np.stack(psd_list, axis=0)

    # Calculate the mean and standard deviation of the PSDs
    plt.semilogy(freq, mean_psd, color='blue', label='Mean PSD')
    plt.scatter(freq[top_peaks], mean_psd[top_peaks], color='red', 
marker='x', s=100, zorder=5)
    plt.title('Power Spectral Density (PSD) with Peaks')
    plt.xlabel('Frequency (Hz)')
    plt.ylabel('Power/Frequency (dB/Hz)')
    plt.grid(True)
    plt.savefig(save_path, format='pdf')
    return maxi


treatments = ["ColumnRough", "ColumnSmooth", "BottomRough", 
"BottomSmooth"]

maxima = []
for treatment in treatments:
    inipath=r"File path to the force measurement data" + treatment
    treatment = os.path.basename(inipath)
    save_path = "G:/My 
Drive/Publication_Nandha/01_Revision_1/PowerSpectralAnalysis/" + 
treatment + "PSD_average.pdf"
    data = LoadFiles(inipath)
    freq_list, psd_list, peaks_list = PSD(data)
    maxi = Plot_PSD_average(freq_list, psd_list, peaks_list, save_path)
    maxima.append(maxi)

for i in maxima:
    dispMaxima(i)

mean_psd=[]
for i in psd_list:
    mean= sum(i)/len(i)
    mean_psd.append(mean)
```

Table S2. Fish catchment data

Available for download at
https://journals.biologists.com/bio/article-lookup/doi/10.1242/bio.060533#supplementary-data

Biology Open • Supplementary information

Table S3. Id number (Id), wet weight (WW), Total length (TL), Body depth (BD), Maximum head width (HW) and fineness-ratio (FR) of the ball and 3D-Plastic fish.

Id	WW [g]	TL [cm]	BD [cm]	HW [cm]	FR	Additional Info
3D_Fish_1	8.13	7.6	1.50	1.43	5.17	Pectoral fins non-spread
3D_Fish_2	7.48	7.3	1.46	1.36	5.17	Pectoral fins spread
3D_Fish_3	15.30	9.3	1.90	1.99	4.76	Pectoral fins non-spread
BALL	14.02	3.0	3.00	3.00	1.00	Sphere shape solid rubber ball

Table S4. Mean hydraulic forces and morphometric data

Available for download at
https://journals.biologists.com/bio/article-lookup/doi/10.1242/bio.060533#supplementary-data

Table S5. The following code is written in python and was used to import data and train and validate a Convolutional Neural Network in Google colab.

```
Its purpose was to classify hydraulic force time series data.
Author: Joschka Wiegleb
```

```
from google.colab import drive
drive.mount("/content/gdrive")

import pandas as pd
import numpy as np
import time
import os
from sklearn.model_selection import train_test_split
import tensorflow as tf
from tensorflow.keras import layers
from keras.models import Sequential
from keras.layers import Dense
from keras.layers.convolutional import Conv1D
from keras.layers.convolutional import MaxPooling1D
from keras.layers import Flatten
from keras.layers import Embedding
from keras.layers import LSTM
from keras.layers import Masking
from keras.layers import GlobalMaxPooling1D
from keras import optimizers
from keras import Input
from keras import Model
from keras import callbacks
from keras.layers import Concatenate, Dense, LSTM, Input, concatenate
from sklearn.preprocessing import MinMaxScaler
from keras.layers import Dropout
import random
import matplotlib.pyplot as plt
import numpy
import math
from random import shuffle
import statistics

import scipy.io

kernel_size = 70 #70
kernel = np.ones(kernel_size) / kernel_size

from itertools import islice

def downsample(rows, proportion):
    return list(islice(rows, 0, len(rows), int(1/proportion)))

def ExtractData(mat, i):
    data = mat.items()
    liste = list(data)
    proportion=0.1 #downsample and collect 10 % of data
    z1=liste[0][1][3,:][0:10000]
    z1=np.convolve(z1, kernel, mode='same')
    z1=downsample(z1, proportion)
    z2=liste[0][1][3,:][10000:20000]
    z2=np.convolve(z2, kernel, mode='same')
```

Biology Open • Supplementary information

```
z2=downsample(z2, proportion)
z3=liste[0][1][3,:][20000:30000]
z3=np.convolve(z3, kernel, mode='same')
z3=downsample(z3, proportion)
z4=liste[0][1][3,:][30000:40000]
z4=np.convolve(z4, kernel, mode='same')
z4=downsample(z4, proportion)
z5=liste[0][1][3,:][40000:50000]
z5=np.convolve(z5, kernel, mode='same')
z5=downsample(z5, proportion)
tx1=liste[0][1][4,:][0:10000]
tx1=np.convolve(tx1, kernel, mode='same')
tx1=downsample(tx1, proportion)
tx2=liste[0][1][4,:][10000:20000]
tx2=np.convolve(tx2, kernel, mode='same')
tx2=downsample(tx2, proportion)
tx3=liste[0][1][4,:][20000:30000]
tx3=np.convolve(tx3, kernel, mode='same')
tx3=downsample(tx3, proportion)
tx4=liste[0][1][4,:][30000:40000]
tx4=np.convolve(tx4, kernel, mode='same')
tx4=downsample(tx4, proportion)
tx5=liste[0][1][4,:][40000:50000]
tx5=np.convolve(tx5, kernel, mode='same')
tx5=downsample(tx5, proportion)

ty1=liste[0][1][5,:][0:10000]
ty1=np.convolve(ty1, kernel, mode='same')
ty1=downsample(ty1, proportion)
ty2=liste[0][1][5,:][10000:20000]
ty2=np.convolve(ty2, kernel, mode='same')
ty2=downsample(ty2, proportion)
ty3=liste[0][1][5,:][20000:30000]
ty3=np.convolve(ty3, kernel, mode='same')
ty3=downsample(ty3, proportion)
ty4=liste[0][1][5,:][30000:40000]
ty4=np.convolve(ty4, kernel, mode='same')
ty4=downsample(ty4, proportion)
ty5=liste[0][1][5,:][40000:50000]
ty5=np.convolve(ty5, kernel, mode='same')
ty5=downsample(ty5, proportion)

if len(liste[0][1][3,:]) > 60000:
    z6=liste[0][1][3,:][50000:60000]
    z6=np.convolve(z6, kernel, mode='same')
    z6=downsample(z6, proportion)
    tx6=liste[0][1][4,:][50000:60000]
    tx6=np.convolve(tx6, kernel, mode='same')
    tx6=downsample(tx6, proportion)
    ty6=liste[0][1][5,:][50000:60000]
    ty6=np.convolve(ty6, kernel, mode='same')
    ty6=downsample(ty6, proportion)
else:
    #fill empty with zeros
    z6=liste[0][1][3,:][50000:]
    print('Length: ',len(z6))
    z6=np.convolve(z6, kernel, mode='same')
    #z6=downsample(z6, proportion)
```

```
        add=10000-len(z6)
        zeros = [0] * add
        zeros=np.asarray(zeros)
        z6=np.concatenate((z6,zeros))
        #z6=np.convolve(z6, kernel, mode='same')
        z6=downsample(z6, proportion)
        tx6=liste[0][1][4,:][50000:]
        tx6=np.convolve(tx6, kernel, mode='same')
        tx6=np.concatenate((tx6,zeros))
        tx6=downsample(tx6, proportion)
        ty6=liste[0][1][5,:][50000:]
        ty6=np.convolve(ty6, kernel, mode='same')
        ty6=np.concatenate((ty6,zeros))
        ty6=downsample(ty6, proportion)

        print('less data than 60000: ',i)


    PrepArray1=[z1, tx1, ty1]
    PrepArray2=[z2, tx2, ty2]
    PrepArray3=[z3, tx3, ty3]
    PrepArray4=[z4, tx4, ty4]
    PrepArray5=[z5, tx5, ty5]
    PrepArray6=[z6, tx6, ty6]
    return PrepArray1, PrepArray2, PrepArray3, PrepArray4, PrepArray5,
PrepArray6


def LoadData_CrossVal(inipath, kernel, treatment):


    inipath=inipath + treatment
    files = os.listdir(inipath)

    dataMaster1=[]
    dataMaster2=[]
    dataMaster3=[]
    pro=0
    for i in files:
        path=os.path.join(inipath,i)
        mat = scipy.io.loadmat(path)
        PrepArray1, PrepArray2, PrepArray3, PrepArray4, PrepArray5,
PrepArray6 =ExtractData(mat,i)
        dataMaster1.append(PrepArray1)
        dataMaster1.append(PrepArray2)
        dataMaster2.append(PrepArray3)
        dataMaster2.append(PrepArray4)
        dataMaster3.append(PrepArray5)
        dataMaster3.append(PrepArray6)
        progress=(pro/len(files))*100
        print('Prgress [%]:', int(progress))
        pro=pro+1

    dataMaster_Master_1=np.asarray(dataMaster1)
    dataMaster_Master_2=np.asarray(dataMaster2)
    dataMaster_Master_3=np.asarray(dataMaster3)

    #create label: Label for coumn smooth is 0
```

Biology Open • Supplementary information

```
    y_1=[]
    y_2=[]
    y_3=[]
    for i in dataMaster_Master_1:
        if treatment == 'ColumnSmooth':
            lab=0
        elif treatment == 'ColumnRough':
            lab=1
        elif treatment == 'BottomRough':
            lab=2
        elif treatment == 'BottomSmooth':
            lab=3
        y_1.append(lab)
        y_2.append(lab)
        y_3.append(lab)

    return dataMaster_Master_1, dataMaster_Master_2, dataMaster_Master_3,
y_1, y_2, y_3


print#combine original data with augmentation
sett1 = np.concatenate((set1, set1_aug, set1_aug2))
sett2 = np.concatenate((set2, set2_aug, set2_aug2))
sett3 = np.concatenate((set3, set3_aug, set3_aug2))

y_sett1 = np.concatenate((y_set1, y_set1_aug, y_set1_aug2))
y_sett2 = np.concatenate((y_set2, y_set2_aug, y_set2_aug2))
y_sett3 = np.concatenate((y_set3, y_set3_aug, y_set3_aug2))


#shuffle data sets randomly

def Shuffle(data, labels):
    sequence = [i for i in range(len(data))]
    shuffle(sequence)

    shuffdata = [data[i] for i in sequence]
    shuffdata=np.asarray(shuffdata)
    shufflabels = [labels[i] for i in sequence]
    shufflabels=np.asarray(shufflabels)
    return shuffdata, shufflabels

Set1, y_Set1 = Shuffle(sett1, y_sett1)
Set2, y_Set2 = Shuffle(sett2, y_sett2)
Set3, y_Set3 = Shuffle(sett3, y_sett3)




import statistics

def ConfusionMatrix(y_pred, y_val):
    #predicted_true
    CS_CS=[]
    CS_CR=[]
    CS_BR=[]
    CS_BS=[]
    CR_CS=[]
```

Biology Open • Supplementary information

```
CR_CR=[]
CR_BR=[]
CR_BS=[]
BR_CS=[]
BR_CR=[]
BR_BR=[]
BR_BS=[]
BS_CS=[]
BS_CR=[]
BS_BR=[]
BS_BS=[]


for i in list(range(0,len(y_pred))):
    max_pred = y_pred[i][0]
    index_pred = 0
    for u in range(1,len(y_pred[i])):
        if y_pred[i][u] > max_pred:
            maxx_pred = y_pred[i][u]
            index_pred = u

    max_val = y_pred[i][0]
    index_val = 0
    for g in range(1,len(y_val[i])):
        if y_val[i][g] > max_val:
            maxx_val = y_val[i][g]
            index_val = g
    if index_pred == index_val and index_pred==3: #BS
        BS_BS.append(1)
    elif index_pred == index_val and index_pred==2: #BR
        BR_BR.append(1)
    elif index_pred == index_val and index_pred==0: #CS
        CS_CS.append(1)
    elif index_pred == index_val and index_pred==1: #CR
        CR_CR.append(1)
    elif index_pred==0 and index_val==1:
        CS_CR.append(1)
    elif index_pred==0 and index_val==2:
        CS_BR.append(1)
    elif index_pred==0 and index_val==3:
        CS_BS.append(1)
    elif index_pred==1 and index_val==0:
        CR_CS.append(1)
    elif index_pred==1 and index_val==2:
        CR_BR.append(1)
    elif index_pred==1 and index_val==3:
        CR_BS.append(1)

    elif index_pred==2 and index_val==0:
        BR_CS.append(1)
    elif index_pred==2 and index_val==1:
        BR_CR.append(1)
    elif index_pred==2 and index_val==3:
        BR_BS.append(1)
    elif index_pred==3 and index_val==0:
        BS_CS.append(1)
    elif index_pred==3 and index_val==1:
        BS_CR.append(1)
```

Biology Open • Supplementary information

```
        elif index_pred==3 and index_val==2:
            BS_BR.append(1)

    CS_CS_I=sum(CS_CS)
    CS_CR_I=sum(CS_CR)
    CS_BR_I=sum(CS_BR)
    CS_BS_I=sum(CS_BS)
    CR_CS_I=sum(CR_CS)
    CR_CR_I=sum(CR_CR)
    CR_BR_I=sum(CR_BR)
    CR_BS_I=sum(CR_BS)
    BR_CS_I=sum(BR_CS)
    BR_CR_I=sum(BR_CR)
    BR_BR_I=sum(BR_BR)
    BR_BS_I=sum(BR_BS)
    BS_CS_I=sum(BS_CS)
    BS_CR_I=sum(BS_CR)
    BS_BR_I=sum(BS_BR)
    BS_BS_I=sum(BS_BS)


    conf=[(CS_CS_I, CS_CR_I, CS_BR_I, CS_BS_I), (CR_CS_I, CR_CR_I,
CR_BR_I, CR_BS_I), (BR_CS_I, BR_CR_I, BR_BR_I, BR_BS_I), (BS_CS_I,
BS_CR_I, BS_BR_I, BS_BS_I)]
    confusionmatrix = pd.DataFrame(conf, columns = ['CS_true' ,
'CR_true', 'BR_true', 'BS_true' ], index=['CS_pred', 'CR_pred',
'BR_pred', 'BS_pred'])
    print('Confusion Matrix:')
    print(confusionmatrix)

    #compute Accuracy
    CS_acc_I=(CS_CS_I/(CS_CS_I+CR_CS_I+BR_CS_I+BS_CS_I))*100
    CR_acc_I=(CR_CR_I/(CS_CR_I+CR_CR_I+BR_CR_I+BS_CR_I))*100
    BR_acc_I=(BR_BR_I/(CS_BR_I+CR_BR_I+BR_BR_I+BS_BR_I))*100
    BS_acc_I=(BS_BS_I/(CS_BS_I+CR_BS_I+BR_BS_I+BS_BS_I))*100
    tot=statistics.mean([CS_acc_I,CR_acc_I,BR_acc_I,BS_acc_I])

    CS_acc = "{:.2f}".format(CS_acc_I)
    CR_acc = "{:.2f}".format(CR_acc_I)
    BR_acc = "{:.2f}".format(BR_acc_I)
    BS_acc = "{:.2f}".format(BS_acc_I)
    total_acc = "{:.2f}".format(tot)

    print('=================================')
    print('Column Smooth accuracy: ', CS_acc, ' %')
    print('Column Rough accuracy: ', CR_acc, ' %')
    print('Bottom Rough accuracy: ', BR_acc, ' %')
    print('Bottom Smooth accuracy: ', BS_acc, ' %')
    print('=================================')
    print('Total model accuracy: ', total_acc, ' %')
    return confusionmatrix, CS_acc, CR_acc, BR_acc, BS_acc



#Cross Validation 1
X_train=np.concatenate((Set1,Set2))
y_train=np.concatenate((y_Set1, y_Set2))
```

Biology Open • Supplementary information

```
X_val=Set3
y_val=y_Set3


#scale data
#X_train = scaler.fit_transform(X_train)
#X_val = scaler.fit_transform(X_val)


from tensorflow.keras.utils import to_categorical
y_train = to_categorical(y_train)
y_val = to_categorical(y_val)



#define Inputs
#input A
n_timesteps, n_features, n_outputs = X_train.shape[2], X_train.shape[1],
X_train.shape[0]
inputA = Input(shape=(n_features, n_timesteps))



#Define Model

from keras.layers import GlobalAveragePooling1D
from keras.layers import Add
from keras.layers import Activation
from keras.layers import BatchNormalization


def ConvModelGlobAvPoolRes(inputA):
    model = Sequential()
    #x = Input(shape=(n_timesteps,n_features))
    x = Conv1D(filters=64, kernel_size=7, activation='relu',
padding='same')(inputA)
    x = Conv1D(filters=64, kernel_size=7, activation='relu',
padding='same')(x)
    #x = Activation('relu')(x)
    x = MaxPooling1D(pool_size=1)(x)
    x_shortcut=x

    x = Conv1D(filters=64, kernel_size=3, activation='relu',
padding='same')(x)
    x = Conv1D(filters=64, kernel_size=3, activation='relu',
padding='same')(x)
    #x = Activation('relu')(x)
    x = MaxPooling1D(pool_size=1)(x)
    x = Add()([x, x_shortcut])

    x = Conv1D(filters=128, kernel_size=3, activation='relu',
padding='same')(x)
    x = Conv1D(filters=128, kernel_size=3, activation='relu',
padding='same')(x)
    #x = Activation('relu')(x)
    x = MaxPooling1D(pool_size=1)(x)
    xx_shortcut=x
```

Biology Open • Supplementary information

```
    x = Conv1D(filters=128, kernel_size=3, activation='relu',
padding='same')(x)
    x = Conv1D(filters=128, kernel_size=3, activation='relu',
padding='same')(x)
    #x = Activation('relu')(x)
    x = MaxPooling1D(pool_size=1)(x)
    x = Add()([x, xx_shortcut])

    x = Conv1D(filters=512, kernel_size=3, activation='relu',
padding='same')(x)
    x = Conv1D(filters=512, kernel_size=3, activation='relu',
padding='same')(x)
    #x = Activation('relu')(x)
    x = MaxPooling1D(pool_size=1)(x)
    xxx_shortcut=x

    x = Conv1D(filters=512, kernel_size=3, activation='relu',
padding='same')(x)
    x = Conv1D(filters=512, kernel_size=3, activation='relu',
padding='same')(x)
    #x = Activation('relu')(x)
    x = MaxPooling1D(pool_size=1)(x)
    x = Add()([x, xxx_shortcut])

    x = Conv1D(filters=1024, kernel_size=3, activation='relu',
padding='same')(x)
    x = Conv1D(filters=1024, kernel_size=3, activation='relu',
padding='same')(x)
    #x = Activation('relu')(x)
    x = MaxPooling1D(pool_size=1)(x)
    xxx_shortcut=x

    x = Conv1D(filters=1024, kernel_size=3, activation='relu',
padding='same')(x)
    x = Conv1D(filters=1024, kernel_size=3, activation='relu',
padding='same')(x)
    #x = Activation('relu')(x)
    x = MaxPooling1D(pool_size=1)(x)
    x = Add()([x, xxx_shortcut])

    x = GlobalAveragePooling1D()(x)
    x = Dense(4, activation="softmax")(x)
    model = Model(inputs=inputA, outputs=x)
    return model


model=ConvModelGlobAvPoolRes(inputA)


#save model
model.save("Path to save the model: Model Name.h5")


#Cross Validation 1 Set 2 and 3
X_train=np.concatenate((Set2,Set3))
y_train=np.concatenate((y_Set2, y_Set3))
```

```
X_val=Set1
y_val=y_Set1


from tensorflow.keras.utils import to_categorical
y_train = to_categorical(y_train)
y_val = to_categorical(y_val)



#define Inputs
#input A
n_timesteps, n_features, n_outputs = X_train.shape[2], X_train.shape[1],
X_train.shape[0]
inputA = Input(shape=(n_features, n_timesteps))
```

Biology Open • Supplementary information

Table S6. Frequency and Power Spectrum Density (PSD) of the five peaks with highest PSD for the different treatments.

Column Rough		Column Smooth	
Frequency [Hz]	PSD [dB/Hz]	Frequency [Hz]	PSD [dB/Hz]
3.91	$8.94 * 10^{-7}$	3.91	$4.83 * 10^{-7}$
15.63	$7.78 * 10^{-7}$	15.63	$4.35 * 10^{-7}$
31.25	$4.64 * 10^{-7}$	31.25	$2.05 * 10^{-7}$
42.97	$2.30 * 10^{-7}$	42.97	$1.86 * 10^{-7}$
148.44	$4.51 * 10^{-8}$	433.59	$1.44 * 10^{-8}$

Bottom Rough		Bottom Smooth	
Frequency [Hz]	PSD [dB/Hz]	Frequency [Hz]	PSD [dB/Hz]
3.91	$4.55 * 10^{-7}$	11.72	$8.41 * 10^{-7}$
11.72	$6.17 * 10^{-7}$	31.25	$1.97 * 10^{-7}$
31.25	$2.93 * 10^{-7}$	42.97	$7.94 * 10^{-7}$
42.97	$1.86 * 10^{-7}$	66.41	$3.88 * 10^{-7}$
386.72	$1.84 * 10^{-8}$	85.94	$3.27 * 10^{-8}$

Biology Open • Supplementary information
